# Usability and Acceptance of an Interactive Tablet-Based Exercise Application: A Mixed Methods Study

**DOI:** 10.3389/fdgth.2020.578281

**Published:** 2020-10-28

**Authors:** Pierrette Baschung Pfister, Bernadette Tobler-Ammann, Ruud H. Knols, Eling D. de Bruin, Rob A. de Bie

**Affiliations:** ^1^Directorate of Research and Education, Physiotherapy Occupational Therapy Research Center, University Hospital Zurich, Zurich, Switzerland; ^2^Nursing and Allied Health Profession Office, Physiotherapy Occupational Therapy, University Hospital Zurich, Zurich, Switzerland; ^3^Department of Epidemiology, CAPHRI Care and Public Health Research Institute, Maastricht University, Maastricht, Netherlands; ^4^University Clinic of Hand- and Plastic Surgery, Department of Hand and Occupational Therapy Research, Inselspital, Bern University Hospital, Bern, Switzerland; ^5^Department of Health Sciences and Technology, Institute of Human Movement Sciences and Sport, ETH Zurich, Zurich, Switzerland; ^6^Division of Physiotherapy, Department of Neurobiology, Care Sciences and Society, Karolinska Institutet, Solna, Sweden

**Keywords:** usability, focus group, resistance training, exercise, training app, telecommunication technologies

## Abstract

**Purpose:** To investigate usability and acceptance of a newly developed interactive, tablet-based exercise application (app) and to explore personal opinions of therapists when using this app in the clinical setting.

**Methods:** Twenty participants (10 therapists and 10 inactive healthy adults) tested usability of this app performing different test tasks, using the think aloud method, and rated overall satisfaction with the System Usability Scale and acceptance with a modified Technology Acceptance Model Questionnaire. For a secondary objective, personal opinions of therapists were evaluated with two focus groups, one for team leaders and one for team members.

**Results:** Overall, the app was judged to be usable. Effectiveness varied between 73 and 90%, overall satisfaction between 70.5 and 85.5/100 points and acceptance between 74 and 80%. Team leader and team member focus groups considered the app as providing a great opportunity for therapy extension, especially because of its blended character. Barriers to its implementation were seen in the existing clinical working processes, personal attitudes of therapists and uncertainty of who would cover expenses for this new form of therapy. Some improvements such as using videos instead of photos, the integration of more interactive tools and the possibility to add additional exercises were suggested in both settings.

**Conclusion:** The app showed high acceptance and usability in trainees and therapists, although some ideas for upgrading functions were formulated. Before this app can be used in clinical practice, feasibility of this blended approach should be evaluated in a clinical setting.

## Introduction

Resistance training (RT) is a crucial element of the general health enhancing physical activity recommendations and guidelines suggest that every adult should perform activities that maintain or increase muscle strength and endurance at least twice a week (1). Such a regular RT program can not only minimize age-related musculoskeletal alterations, thus reducing their impact on health and the aging process, but also improve physical and mental health, as well as quality of life (2, 3). In addition, RT has potential in the prevention and management of several chronic diseases (4, 5). There is broad evidence that RT increases muscle strength, reduces pain and improves functional ability in patients suffering from chronic low back pain, knee osteoarthritis, chronic tendinopathy and in those recovering after hip replacement surgery (6). RT may also improve health-related quality of life in patients with rheumatic diseases (7) and has positive effects on muscle strength and functional outcomes related to mobility in patients with Parkinson's Disease (8). Furthermore, RT enhances physical functioning and reduces the risk of condition-related lymphedema in patients with breast cancer (9), and can also improve multi-dimensional function, pain, tenderness, and muscle strength in women with fibromyalgia (10).

Despite these positive effects of RT, it remains difficult to motivate inactive individuals to participate regularly in physical exercise (11). For (chronic) patients the barriers to starting exercising may be even higher. Physical deconditioning discourages patients from exercising, which in turn worsens the overall deconditioning and creates a vicious cycle (12). The use of telecommunication technologies (TT)—for example the internet, software applications or SMS messaging–may help to promote health behavior change (13, 14). These technologies have the advantage of implementing different persuasive features that may help exercise programs to be more enjoyable and, thereby, enhance motivation to exercise on a regular basis. Examples of these persuasive features include personalisation, self-monitoring, tailoring, goal-setting and comparison through positive and negative reinforcement in the development of physical exercise programs (15, 16). Evidence shows that web-based compared to non-web-based interventions are more effective in achieving behavioral change (e.g., increased exercise time) (17) and in improving home exercise adherence for people with musculoskeletal conditions (18).

One of the disadvantages of using TT for home exercises is the lack of face-to-face contact with a professional, which may reduce adherence to training (19). Several studies highlight the importance of personal support when exercising, both in disease prevention and management (20–22). Via such personal contact, the definition of personalized goals, the consideration of individual fitness levels and disabilities within the exercise prescription, and necessary exercise adaptations are all feasible.

Therefore, an interactive, tablet-based exercise app (called “Fit”) was developed. The aim of this exercise app is to assist and monitor physical exercise novices during an individually tailored home-based progressive resistance training program with remote support. This interactive approach not only allows additional supervision, but also combines the advantages of new technologies with the personal support of a health professional.

Although it is well-known that convincing system designs are crucial for adherence to web-based interventions, the usage of interactive health care applications has often been hampered by their poor design (23). Consequently, usability testing is an essential step for developing usable and enjoyable products and for identifying flaws in an early system‘s design (24, 25). Usability is defined as the extent to which a product can be used by predefined users to achieve particular goals with effectiveness, efficiency and satisfaction in a specific context of use (26). Therefore, it is necessary to perform an exploratory study to test usability of this newly developed interactive, tablet-based exercise app in untrained healthy individuals, prior to exploring its feasibility with patients in clinical practice (27). However, for a newly developed device to be successfully implemented, not only the opinions of actual end-users (individuals who want to become fitter) but also those of the actual health care providers, are crucial to such exploration. As a result, the primary objective of this study was to test usability and acceptance of the Fit app both with physio- and occupational therapists and untrained individuals. In order to increase acceptability of this novel tablet-based Fit app in therapists, who will introduce this app to their patients, the secondary objective of this study was to explore personal opinions of therapists when using it in a clinical setting.

## Materials and Methods

### Study Design

**Figure 1:**
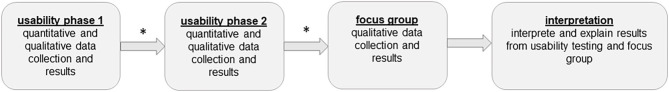
Explanatory design. *after each usability phase the app was revised based on the feedbacks from the participants.

This mixed method study consisted of a usability part followed by a focus group part. Usability was tested in two consecutive phases. After each phase the app was revised based on the feedbacks from the participants (Figure 1).

### Participants and Recruitment

For the usability part, a sample of 20 participants was recruited (ten therapists and ten trainees). The therapists were recruited by means of an explanatory leaflet distributed at the University of Applied Science, Winterthur and the University Hospital Zurich. Therapists' inclusion criteria were that they were (i) a physiotherapist or occupational therapist and (ii) fluent in German. The healthy individuals were recruited from the social environment of the study team members. Inclusion criteria were (i) feeling healthy by self-report; (ii) no currently ongoing treatment by a physician at the time of study; (iii) able to walk independently without a walking aid; (iv) engaging in no more than one exercise session per week; (v) fluent in German. Persons with any potential health risks associated with exercise (assessed with the Physical Activity Readiness Questionnaire (28) were excluded.

For the qualitative section, an invitation to participate in a focus group interview was e-mailed to all therapists from the Department of Physiotherapy and Occupational Therapy of the USZ. The aim was to recruit two therapists (one team leader and one team member) per unit (e.g., neurology, rheumatology, hand therapy) to achieve a broad variety of work settings and patients.

The study protocol was approved by the Research Ethics Committee of ETH Zurich, Switzerland (protocol number EK 2017-N-27) and conformed to the Declaration of Helsinki. All participants signed an informed consent declaration before study entry.

### Rationale of the Sample Size

Successful Identification of key usability problems depends on the number of users included (25). Early studies reported that five evaluators found about two third of all usability problems (29, 30), whereas more recent literature supports the 10±2 rule (31). With ten users a minimum 80% of the problems related to system use may be identified (32). Therefore, we decided to test usability of this exercise app with ten therapists and ten trainees.

For semi-structured focus group discussions, 4–12 participants are recommended for specific topic exploration (33). Less than four people is too few to be considered a “group,” whereas more than 12 is too many persons to allow equal participation in the discussion. The group members usually share certain characteristics (e.g., their profession, social background etc.) to allow a discussion on the same level, and should show interest in the chosen topic (34). It is further recommended to conduct at least two focus group discussions to cover different aspects of the chosen topic (35). Therefore, we invited seven team leaders (TL) and seven team members (TM) per focus group, each representing a therapy unit of the USZ.

### Exercise App

The interactive, tablet-based exercise app was developed by scientist of the ETH, the USZ and Divdiat AG (Dividat Fit, Schindellegi, Switzerland, 93/42/EWG certified). This exercise app provides an interactive, tablet-based, progressive RT programme. “Interactive” means that the supervising therapists design the exercise program individually together with each client (hereafter called trainee) and monitor training progress remotely. During the exercise program, trainees monitor their performed exercises, while the therapists supervise and, as necessary, adapt the exercise's level via remote monitoring.

The exercise app consists of two different parts: “manager” and “play.”

“Manager” is the part where therapists record trainees, compile the exercise program for them and monitor their performed training. It is not accessible to the trainees. Based on trainees' needs, an individually tailored exercise program can be designed and adapted to their skills. For this purpose, a pool of different single- or multiple-joint exercises, each of them at different difficulty levels, is available. For each exercise, individual training parameters (amount of series and repetitions, duration of rest between series, and target intensity using the 6–20 Borg rating of perceived exertion scale (36) can be defined. If needed, a written comment can be added by the therapists. Additionally, therapists have the option of choosing if trainees should rate their current level of pain and mood on a visual analog scale from 0 to 10. Once all exercises are selected and training parameters defined, the desired order of the exercise must be determined. This is important, as the app guides trainees step-by-step through the individually adjusted exercise program. As soon as the trainees have performed the first training session, the therapists can see a graphical and tabular overview of the performed training within “manager.”

**Figure 2:**
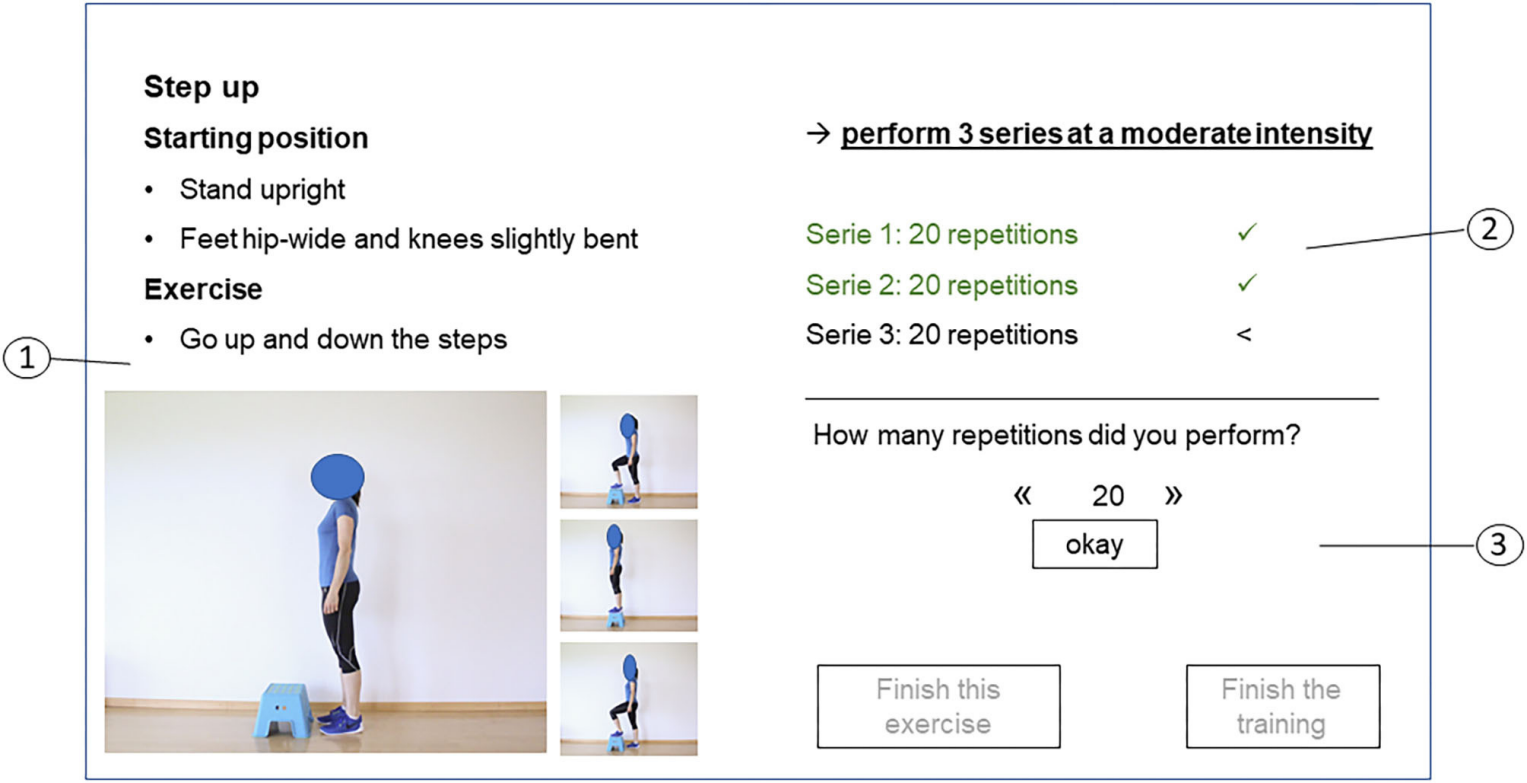
Screen with exercise description of “play.” 1: Exercise overview presenting the exercise description (1), the target training parameters (series, repetitions and intensity) (2) and the possibility to indicate performed training parameters and to finish the exercise or the training (3).

“Play” is the part where trainees can see their exercise program and record their performed exercises. After finishing each exercise, trainees have to record perceived exertion (rating of perceived exertion scale from 6 to 20) for it as well as possible pain on a visual analog scale from 0 to 10. Subsequently, they receive automatically generated feedback corresponding to their reported training intensity, as follows: (1) perceived exertion score is in the predefined level: a positive response is given; (2) perceived exertion score is higher than the predefined level: trainees are suggested to perform the exercise at a lower level; (3) perceived exertion score is lower than the predefined level: trainees are motivated to increase volume or intensity of the exercise; (4) pain is recorded: trainees are advised to perform the exercise correctly and to contact their therapists. In addition to this feedback, the app provides a short statement about the advantages of exercise or a motivational quotation. Additionally, trainees have the option to write a brief note to their therapists. Only after having recorded all training parameters is the next exercise provided. At the end of a training session, trainees receive an overview of their achieved training results. Figure 2 illustrates a screen with the exercise description, the target training parameter and the option to indicate performed repetitions and series of an exercise.

### Procedures

#### Usability

**Figure 3:**
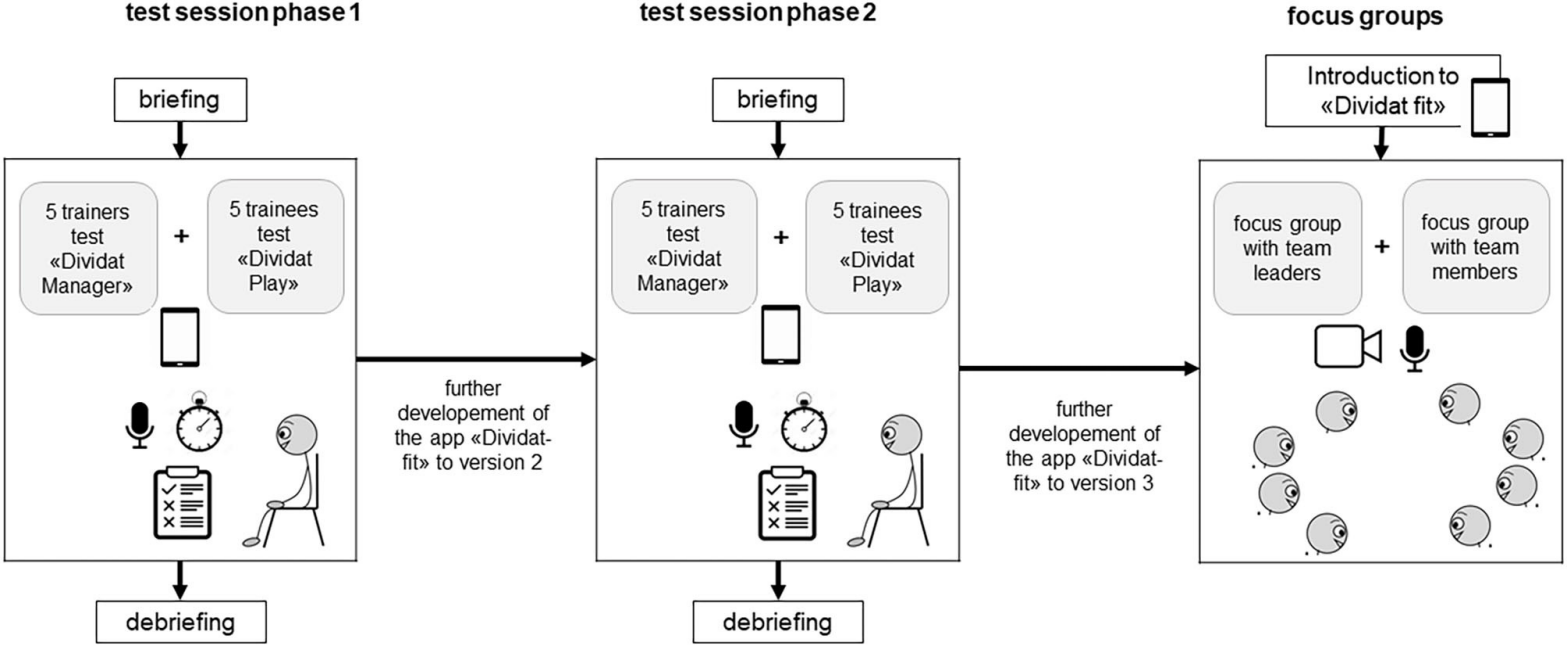
Usability procedure.

Usability was determined by using the separate feedback from two target stakeholder populations: from the perspective of supervising therapists the “manager” component of the app was assessed, while inactive healthy adults tested usability of “play” (Figure 3). In both phases, the two stakeholder populations tested usability separately by doing different test tasks categorized into four and five topics, respectively (Tables 1, 2). The test procedure was organized in two phases. In phase one, five therapists and five trainees tested usability. Based on their feedback, adaptations to “manager” and “play” were implemented. In phase two, five new therapists and five new trainees tested usability of the adapted “manager” and “play” and provided feedback on usability. Based on this feedback, further adjustments were then made.

**Table 1:** Tasks designed for the Therapists.

**Topics and respective tasks**		**Estimated time for a topic**
**Topic I: register trainees**		5 min
Task 1:	Enter name of trainees	
Task 2:	Enter birthdate of trainees	
**Topic II: create an individual exercise program**		20 min
Task 3:	Choose trainee from the list of trainees	
Task 4:	Open standard exercise program	
Task 5:	Change this program into an individual exercise program	
Task 6:	Determine order of the exercises	
**Topic III: check training diary**		10 min
Task 7:	Choose trainee from the list of trainees	
Task 8:	Open training diary	
Task 9:	Interpret figure and table of the training diary	
**Topic IV: adapt individual exercises of an existing exercise program**		5 min
Task 10:	Add a new exercise	
Task 11:	Change an existing exercise	
Task 12:	Adapt repetitions and series and target intensity	
Task 13:	Write an individual comment	

**Table 2:** Tasks designed for the Trainees.

**Topics and respective tasks**		**Estimated time for a topic**
**Topic I: start exercise program**		2 min
Task 1:	Activate the tablet	
Task 2:	Start the exercise program	
Topic II: view first exercise^*^		3 min
Task 3:	Read instruction of the first exercise	
Task 4:	Click on the pictures to see the correct performance	
Task 5:	Read the target training parameters (repetitions, series, break, intensity)	
Topic III: record training diary^*^		3 min
Task 6:	Record performed repetitions and series	
Task 7:	Complete first exercise	
Task 8:	Record intensity and if required pain of the first exercise	
Task 9:	Write a comment	
Topic IV: automatically generated feedbacks^*^		3 min
Task 10:	Read the automatically generated feedback, the motivation or information citations and the overview of the performed exercise.	
**Topic V: check training diary**		3 min
Task 11:	Examine and interpret the training diary	

* *Topic 2–4 were repeated for every four exercise*.

The usability testing took place in a laboratory setting which was equipped with all necessary requirements (e.g., tablet, audio-recording system) (Figure 3). The test scenario consisted of a *briefing*, the *test session* itself and a *debriefing*.

1. The *briefing* contained the introduction to the test session process and the applied questionnaires.
2. The *test session* contained different elementary tasks for the app's usage (Tables 1, 2). Participants independently completed these tasks in a predefined sequence, while communicating when they started and finished a task. While participants performed the tasks, they were encouraged to verbalize their thoughts, and these were audio-recorded (37). The test moderator recorded the time required to complete the task and checked results after task completion. During the whole session, the test moderator sat in the same room, but out of sight of the participant, to limit interaction between moderator and participant. The moderator only intervened when the participant was unable to complete a task. After finishing all tasks, the participants filled out the “System Usability Scale” (SUS) (38, 39) and a modified version of the “Technology Acceptance Model” (TAM) questionnaire.
3. The test scenario ended with a *debriefing* consisting of a short discussion and subsequent discharge.

#### Focus Groups

The focus group interviews took place at the USZ. One focus group was organized for the TL and another for the TM, each lasting 90 min (Figure 3). Before starting with the focus group discussion, the co-moderator presented the app to the therapists by means of a power point presentation. A tablet was used to illustrate the app, which no participants had seen beforehand. The interviews were audio-recorded and filmed. As proposed by Pelz et al. (34), the moderator led the discussion, while the co-moderator took notes on flipcharts. These notes served as a summary of what has been said during the discussion.

## Outcome Measurements

### Usability (Effectiveness, Efficiency and Satisfaction) and Acceptance

For *effectiveness* and *efficiency* of the FIT app, measurements of performance (task completion, errors, time needed) were recorded by the moderator (40), together with reasons for failed completion and relevant comments from the “think aloud” method (24).

For overall *satisfaction*, the System Usability Scale (SUS) was used (38, 39). The SUS includes 10 items about several aspects of usability, such as ease of use or complexity. Each item can be scored on a 5 point Likert scale from 1 (strongly disagree) to 5 (strongly agree). The SUS total score ranges from 10 to 100, higher scores indicating better usability.

For *acceptance*, a modified version of the Technology Acceptance Model (TAM) questionnaire (41) was used. The modified TAM consists of 20 items which can be rated on a 7 point Likert scale ranging from 1 (strongly disagree) to 7 (completely agree), together with an open question about desired additional options for the app. The items are divided into four subgroups: perceived usefulness (7 items), perceived ease of use (6 items), attitude toward using (4 items), and intention to use (3 items). The scores of the subgroups range from 1 to 7 and the total score from 20 to 140.

All outcome measurements and the way they were measured and analyzed are summarized in Table 3.

**Table 3:** Outcome measurements usability.

**Outcome**	**How was it measured and analyzed?**
Effectiveness	Quantitative: • Task completion rateQualitative: • Reasons for failed completion • Description of errors • Comments from the think aloud method
Efficiency	Time needed to complete a task • Number of tasks that could be completed within the given time limits
Satisfaction	System Usability Scale • Mean, standard deviation and minimum/maximum of the total sore
Acceptance	TAM • Mean, standard deviation and minimum/maximum of the total sore and the four subscales

### Focus Group Interview Guide

The interview guide for the focus group discussion comprised four pre-defined questions, around which the discussion was shaped. These were:

1. What are your spontaneous thoughts after the presentation of the app?
2. How could you (not) envisage using this app for your patients?
3. Do you feel this app could influence training motivation in your patients?
4. What would you need/already have at your workplace for a smooth integration of this app in your clinical practice?
5. Can you describe your attitude toward using such exercise apps during your daily work?

### Data Analysis

#### Usability

Descriptive statistical analysis of outcome measurements was done using the software IBM SPSS statistics for windows version 22.0 (Armonk, NY, USA). Effectiveness consisted of the binary (yes/no) task completion rate (proportion of participants that completed a task correctly) and the errors detected. Additionally, participants' reasons for failed completion, together with the comments from the think aloud method, were also reported. Efficiency was rated as high when all tasks within a topic could be performed within the given time limit. For the total score of the SUS and the TAM, all item scores were summed. Additionally, the four subscales of the TAM were scored using the mean value of the respective item responses. Then the mean, standard deviation and minimum/maximum values of the total score and the subscales were calculated.

#### Focus Group Interviews

As this study aimed to summarize a broad variety of opinions rather than focusing on group interactions or single statement (“who said what and how”), the “Focus group Illustration Maps” (FIMs) method was adopted to analyse the interviews (34). The following steps of analysis were carried out:

1. Transcribing the flipchart notes from the focus group interviews in a preliminary FIM.
2. Listening to the audio-recording of the focus group discussion; incorporating statements in the preliminary FIM.
3. Re-listening to the audio-recording, then checking the FIMs for accuracy and completeness.
4. Grouping, organizing and giving “weight” to the correlations between the different statements.
5. Finalizing the FIMs by re-listening to the audio-recording again.
6. E-mailing the FIMs to the participants for member checking.
7. Implementing feedback from member checking.
8. Merging FIMs from therapists and leaders.
9. Finalizing the FIMs; translation into English language.
10. Writing the report.

## Results

**Figure 4:**
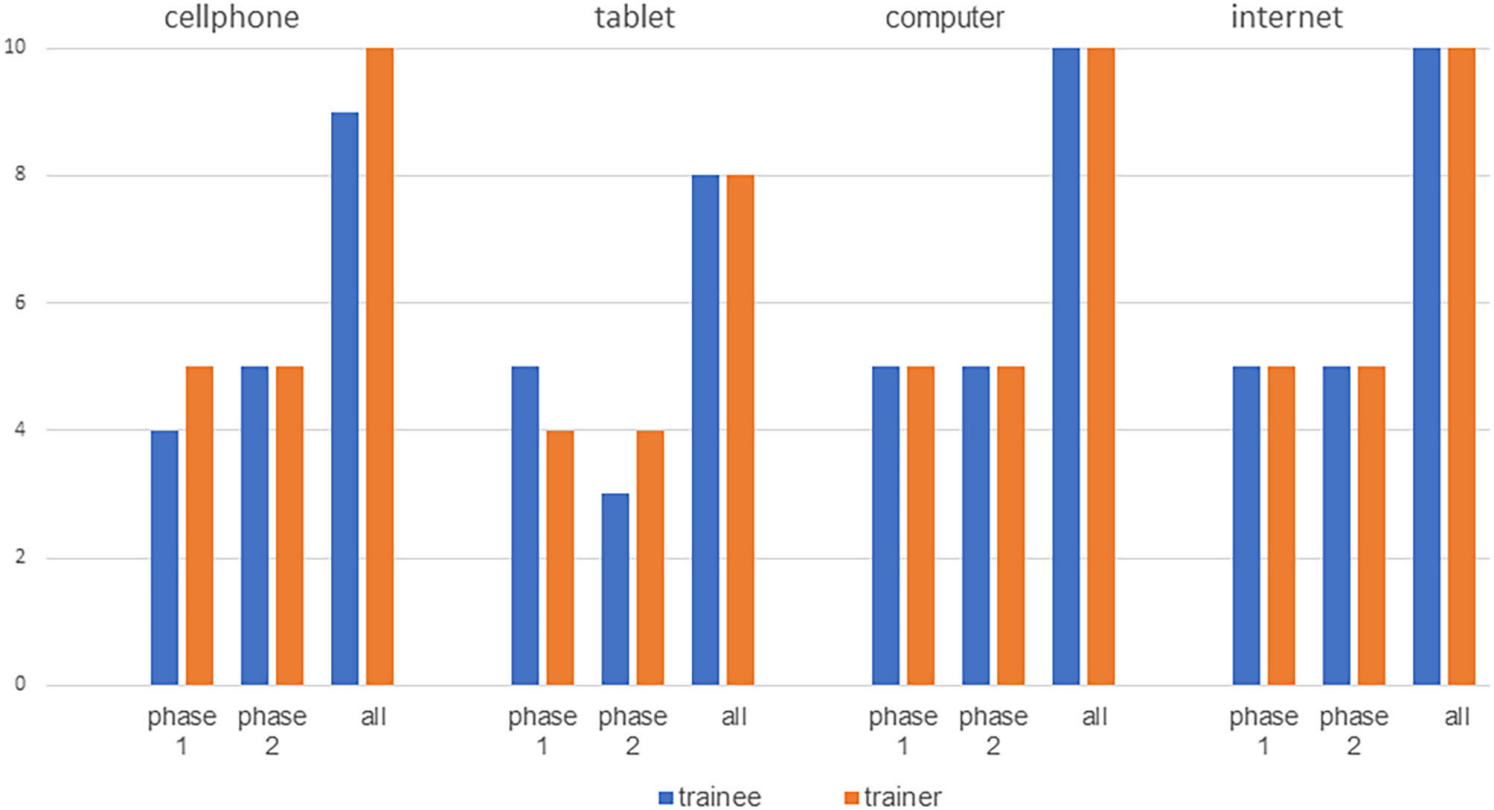
Familiarity with technology.

For usability testing, 20 participants (10 therapists and 10 trainees) were consecutively recruited. Demographic data are presented in Table 4. Most therapists as well as trainees indicated being familiar with the internet, computer, tablets or cell phones (Figure 4). In the focus group interviews, seven TL and five TM participated. Eight participants (6 TL and 2 TM) had a Master's degree and four (1 TL and 3 TM) a Bachelor's degree. Five participants (3 TL and 2 TM) described personal use of exercise apps and three of them (1 TL and 2 TM) indicated using exercise apps in their clinical settings. The remaining seven participants did not use apps at all. Demographic data are presented in Table 5.

**Table 4:** Demographic data of usability participants.

	**Trainees (*n* = 10)**	**Therapists (*n* = 10)**
Gender female/male		
All	7/3	8/2
Phase 1	3/2	5/0
Phase 2	4/1	3/2
Age in years mean (SD)		
All	57 (10)	38 (9)
Phase 1	59 (13)	38 (12)
Phase 2	55 (8)	38 (3)
BMI mean (SD)		
All	26.2 (4.7)	23.6 (3.6)
Phase 1	24.0 (3.1)	22.8 (4.0)
Phase 2	28.4 (5.3)	24.5 (3.3)
Professional experience in years mean (SD)		
All		10 (7)
Phase 1		12 (10)
Phase 2		9 (4)

*BMI, body mass index; SD, standard deviation*.

**Table 5:** Demographic data of focus group participants.

	**Team members** **(*n* = 5)**	**Team leaders** **(*n* = 7)**
Gender female/male	2/3	6/1
Age in years mean (SD)	32 (5)	46 (8)
Professional experience in years mean (SD)	7 (5)	20 (8)
Percentage of employment mean (SD)	82 (4)	83 (13)
Work time dedicated for patients in % mean (SD)	80 (7)	36 (19)

*n, number, SD, standard deviation*.

### Usability

#### Effectiveness

Tasks completion rate, reasons for failed completion, errors and comments are presented in Table 6 for therapists and in Table 7 for trainees. In both phases, the trainees needed a short period of instruction before starting with the test tasks.

**Table 6:** Tasks completion rate, reasons for failed completion, errors and comments of the therapists.

	**Phase 1**			**Phase 2**		
**Topic**	**Tasks completion rate (reason for failed completion)**	**Errors**	**Comments**	**Tasks completion rate (reason for failed completion)**	**Errors**	**Comments**
Register new trainees	Task 1: 100% Task 2: 0% (unfamiliar format of birth date)			Task 1: 100% Task 2: 100%		
Create an individual exercise program	Task 3: 100% Task 4: 100% Task 5: 100% Task 6: 100%	• Therapists did not see the photos and description of the exercises • Some instructions were written in English (even though the program was in German) • Values higher than 20 could be recorded for the 9–20 Borg-scale • In the input window the series and breaks were numbered continuously, although only the series should be numbered • The exercises were numbered, but this number had no meaning		Task 3: 100% Task 4: 100% Task 5: 100% Task 6: 100%	• The first digit of the repetitions could not be deleted • The order of the training parameters was not consistent within the different exercises • When a new exercise was added to the exercise program, it appears in the first place instead of at the end of the exercise program	• “*I would delete all the exercises and then make a new program”* • “*I would like to have my own standard program”* • “*The possibility to copy the amount of repetitions and series would be great….but otherwise it is a useful design”* • “*A drag and drop button would be nice”*
Check a training diary	Task 7: 100% Task 8: 100% Task 9: 0% (figures and tables were too crowded and confusing)	• The order of the list with the participants could not be changed	• “*The figures and tables are really not intuitive and self-explaining*” • “*They [the figures] are totally useless*” • “*This [the figures] is not readable, there is one line upon the other*”	Task 7: 100% Task 8: 100% Task 9: 60% (no scales for the x- y-axes, colors of the lines were not distinguishable from each other)		• “*This is not clear, the colors of the lines are confusing”* • “*I think this should be a target-performance comparison”* • “*There are too many lines, it is not obvious which line represents what”*
Adapt individual exercises	Task 10: 100% Task 11: 100% Task 12: 100% Task 13: 100%	–	–	Task 10: 100% Task 11: 100% Task 12: 100% Task 13: 100%	–	–

**Table 7:** Tasks completion rate, reasons for failed completion, errors and comments of the trainees.

	**Phase 1**			**Phase 2**		
**Topic**	**Task completion rate (reason for failed completion)**	**Errors**	**Comments**	**Task completion rate (reason for failed completion)**	**Errors**	**Comments**
Start exercise program	Task 1: 100% Task 2: 100%	–	–	Task 1: 100% Task 2: 100%	–	
View first exercise	Task 3: 100% Task 4: 100% Task 5: 100%	–	–	Task 3: 100% Task 4: 100% Task 5: 100%	–	• “*It would be nice to have videos instead of photos”*
Record training diary	Task 6: 80% (it was not clear where to report the performedrepetitions) Task 7: 100% Task 8: 60% (very low sensitivity of the touch screen forthe Borg-Scale) Task 9: 80%	• No possibility to add series • No “return” button • No text was seeable while writing a note	• “*Where can I click?”* • “*It is not clear where it is possible to insert something”* • “*When I was too fast: is there no “return” button?”* • “*I hoped I could see anywhere what I‘m writing*”	Task 6: 100% Task 7: 100% Task 8: 100% Task 9: 100%	–	–
Automatically generated feedbacks	Task 10: 60% (screen disappeared too fast)	–	• “*oh, that's too fast”*	Task 10: 100%	–	• “*It‘s cool to have these feedbacks”*
Check training diary	Task 11: 0% (screen with the training diary was very crowded and disappeared after a few seconds)	• Figure could be enlarged with two digits but then not minimized to the original size • Axis were not labeled	–	Task 11: 60% (axis were not clearly labeled)	–	• “*I normally like statistics and figures, but here I needed help”*

#### Efficiency

In both phases, therapists and trainees could fulfill all but one task within the estimated time limit. For therapists, it was task 9 “*interpret figure and table of the training diary*,” which was problematic, while for trainees it was task 11 “*examine and interpret the training diary for trainees.”* Therefore, the topic “*check training diary”* could not be completed within the predefined timeframe.

#### Satisfaction

Therapists from phase one rated satisfaction with the app higher than those from phase two, whereas the reverse was the case in trainees (Table 8).

**Table 8:** System Usability Scale (SUS).

	**Phase 1**		**Phase 2**	
	**Mean (SD)**	**Min/max**	**Mean (SD)**	**Min/max**
Trainees	70.5 (11.1)	52.5/80.0	81.5 (12.8)	67.5/97.5
Therapists	85.5 (10.5)	67.5/92.5	73.5 (10.8)	62.5/87.5

#### Acceptance

The TAM included one question about the usefulness of videos, which was redundant, as there were no videos integrated in the app. Additionally, the two questions about the ease of use of photos could not be answered by the therapists, as there were no photos integrated in the “manager.” Thus, the possible maximum scores for trainees and therapist were 133 and 119 respectively. The percentage of the total score was computed to allow comparison of results between trainees and therapists (Table 9).

**Table 9:** Technology Acceptance Model (TAM).

		**Phase 1**		**Phase 2**	
		**Mean (SD)**	**Min/max**	**Mean (SD)**	**Min/max**
Trainees	Perceived usefulness	5.5 (0.5)	4.6/5.9	6.6 (0.4)	6.0/7.0
	Perceived ease of use	6.0 (0.2)	5.8/6.4	6.1 (1.1)	4.4/7.0
	Attitude toward using	6.4 (0.5)	5.8/7.0	6.3 (0.7)	5.5/7.0
	Intention to use	5.7 (0.4)	5.3/6.3	6.2 (0.8)	5.3/7.0
	Total score (max: 133)	111 (4)	106/115	120 (12)	104/133
	Total score (%)	84 (3)	80/86	90 (9)	78/100
Therapists	Perceived usefulness	6.1 (0.5)	5.4/6.6	5.9 (0.5)	5.4/6.8
	Perceived ease of use	5.4 (1.4)	3.0/6.6	5.8 (0.9)	4.6/6.6
	Attitude toward using	6.4 (0.9)	4.8/7.0	5.9 (0.9)	4.8/7.0
	Intention to use	6.2 (0.6)	5.3/7.0	5.3 (1.0)	4.0/6.7
	Total score (max: 119)	101 (12)	88/114	97 (14)	83/115
	Total score (%)	85 (10)	74/96	81 (11)	70/97

Therapists as well as trainees identified several ideas for improvements for the app. For the “manager” therapists proposed (i) the possibility to filter the exercises' overview for individual use, (ii) the inclusion of videos instead of photos, (iii) other exercises and help functions and (iv) other training parameters, e.g., heart rate or blood pressure. For the “play,” the trainees proposed (i) help functions, (ii) exercise instruction by videos instead of photos, (iii) return buttons and (iv) the possibility to contact the therapists via e-mail.

### Focus Groups

#### Usefulness

**Figure 5:**
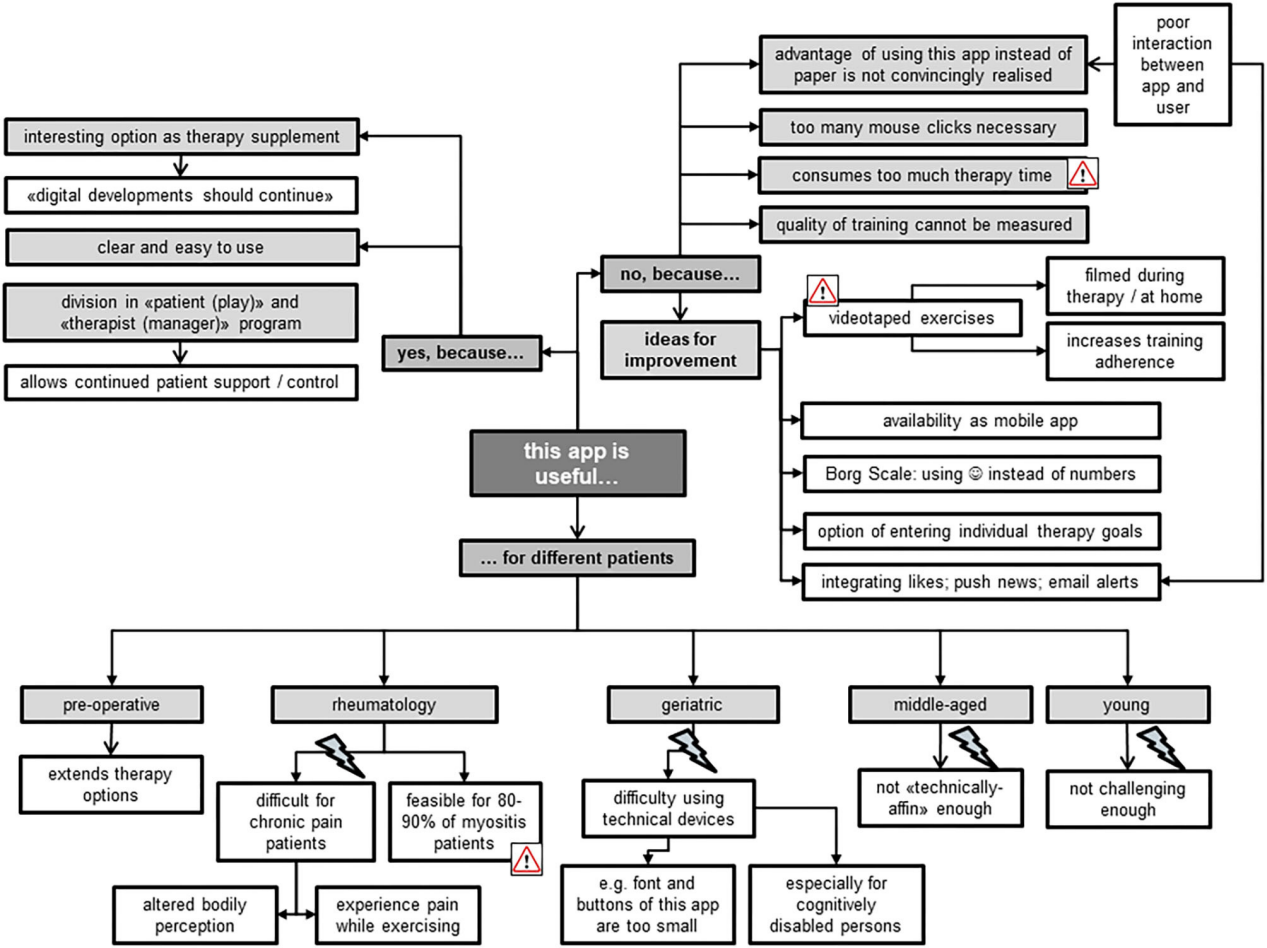
Therapists' perceived usefulness of the Fit app for daily work.

In general, both TL and TM perceived the Fit app as a useful therapy supplement which they could contemplate introducing to different patient groups (Figure 5). Aside from certain specific reservations for its use—e.g., for “*technically disinclined patients*” —they believed that up to 90% of myositis patients could benefit from this app. The focus group members highlighted that most of these patients traveled from all over Switzerland to the USZ for single therapy sessions. Therefore, use of the app could increase training intensity, as patients could work from home, while simultaneously being offered professional assistance from the USZ thanks to its division into “manager” and “play” components. Both TL and TM liked the need of the presence of an expert–the therapist–which was seen as an important difference when compared with other fitness apps without a “professional” feedback function. The use of “blended therapy” increased the acceptability of the app among the focus group members, because it reduced their fear of being replaced by technical applications. Particularly for TM, it was seen as important that use of the app should not consume the whole therapy time, but rather serve as part of a therapy session alongside “face-to-face” treatment. During the interview, it became evident that all members would have preferred the use of videos instead of pictures to illustrate the exercises, because “*everyone likes watching videos.”* Further ideas for improvement mostly aimed at increasing the app's advantages over the common use of hardcopies to illustrate the prescribed exercises (as “*stick figure”*). Other provided examples included integrating push news as a memory function to contact the patient, and the app's availability via smartphone rather than tablet-computer.

#### Training Motivation

**Figure 6:**
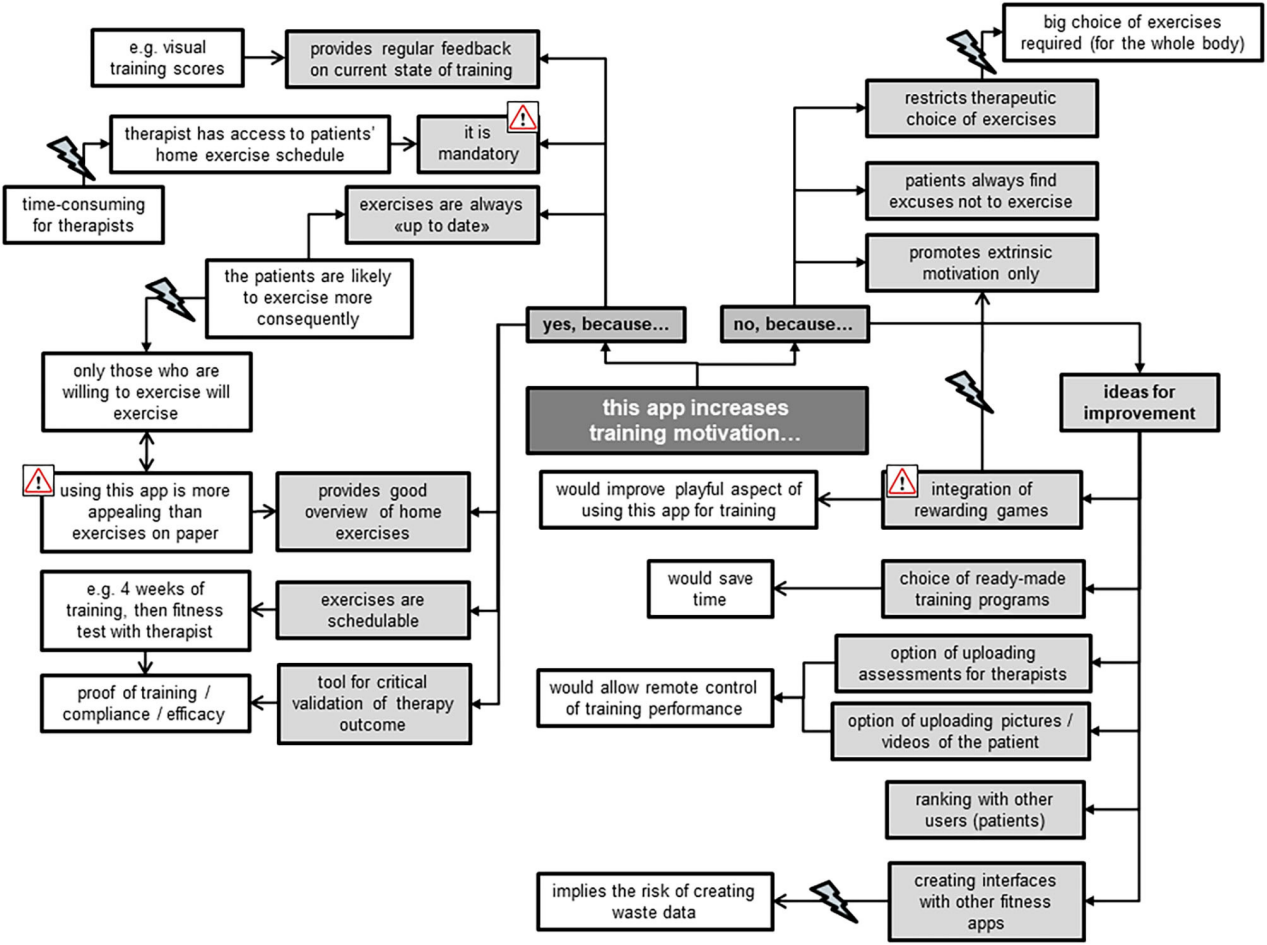
Therapists' statements about perceived training motivation when using the Fit app for their patients.

The TL all agreed on the app's feasibility for increasing training motivation in comparison with the current wide use of hardcopies to present home exercise programs (Figure 6). For example, they emphasized the app's positive commitment to training due to its active therapist involvement in support and control of progress of patients' home exercises. However, they imagined this involvement as rather time-consuming for their already very busy therapists. They further liked the app as a tool of evidence for the effectiveness of therapy thanks to its immediate provision of training progress. In comparison to the TL, the TM took a more critical stance toward the app's ability for increasing training motivation. They doubted that patients would increase their exercise volume when using the app instead of hardcopy exercise schedules, and were reluctant to promote extrinsic rather than intrinsic training motivation. In addition, they feared being restricted in their choice of exercises to design individually adjusted exercise programs when using the preset exercises of the app. Ideas for improvements to increase training motivation when using this app were plentiful (Figure 6). The focus group members, however, did not reach consensus regarding whether implementation of rewarding games would really increase training motivation. Some stated that rewarding games are no valuable feedback for training quality, but rather trigger extrinsically motivated training aspects, e.g., to please the therapist. Others missed the playfulness of gaming when using the app for training at home, and advocated a bonus when exercising, emphasizing “*everybody likes collecting things.”*

#### Smooth Integration

**Figure 7:**
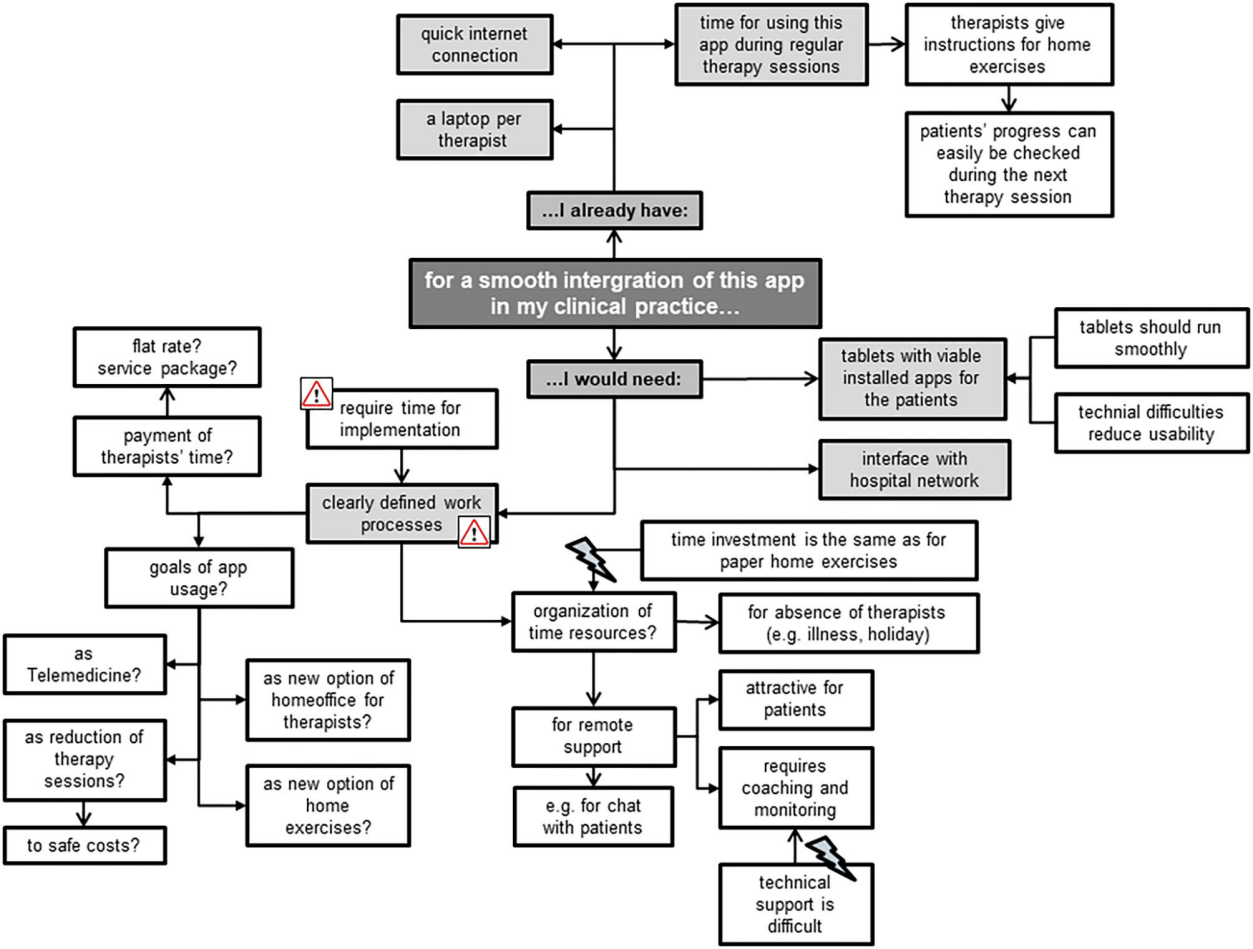
Workplace requirements for a smooth integration of the Fit app in clinical practice.

The focus group members mainly mentioned environmental factors that could facilitate a smooth integration of the Fit app into their clinical practice, e.g., a laptop per therapist (Figure 7). Most of the TM appreciated the app's potential for remote support, but had reservations regarding personal time resources for coaching and monitoring patients. The TL, however, put more emphasis on the uncovered costs and missing work processes involved in implementing the app. Some of the latter feared that this innovation is “*just one more thing I have to do,”* expressing reservations for its use, while some of the former stated that “*having no time”* is not an excuse for non-usage. Other concerns mentioned included the fear of not being able to address patients' technical problems when using this app and when precisely to use it who best to use it for. Besides rising concerns, the focus group members also created new ideas for this app's usage, e.g., as an option for Telemedicine (“call center”) and the related possibility for therapists to do more home-office working.

#### Attitude

**Figure 8:**
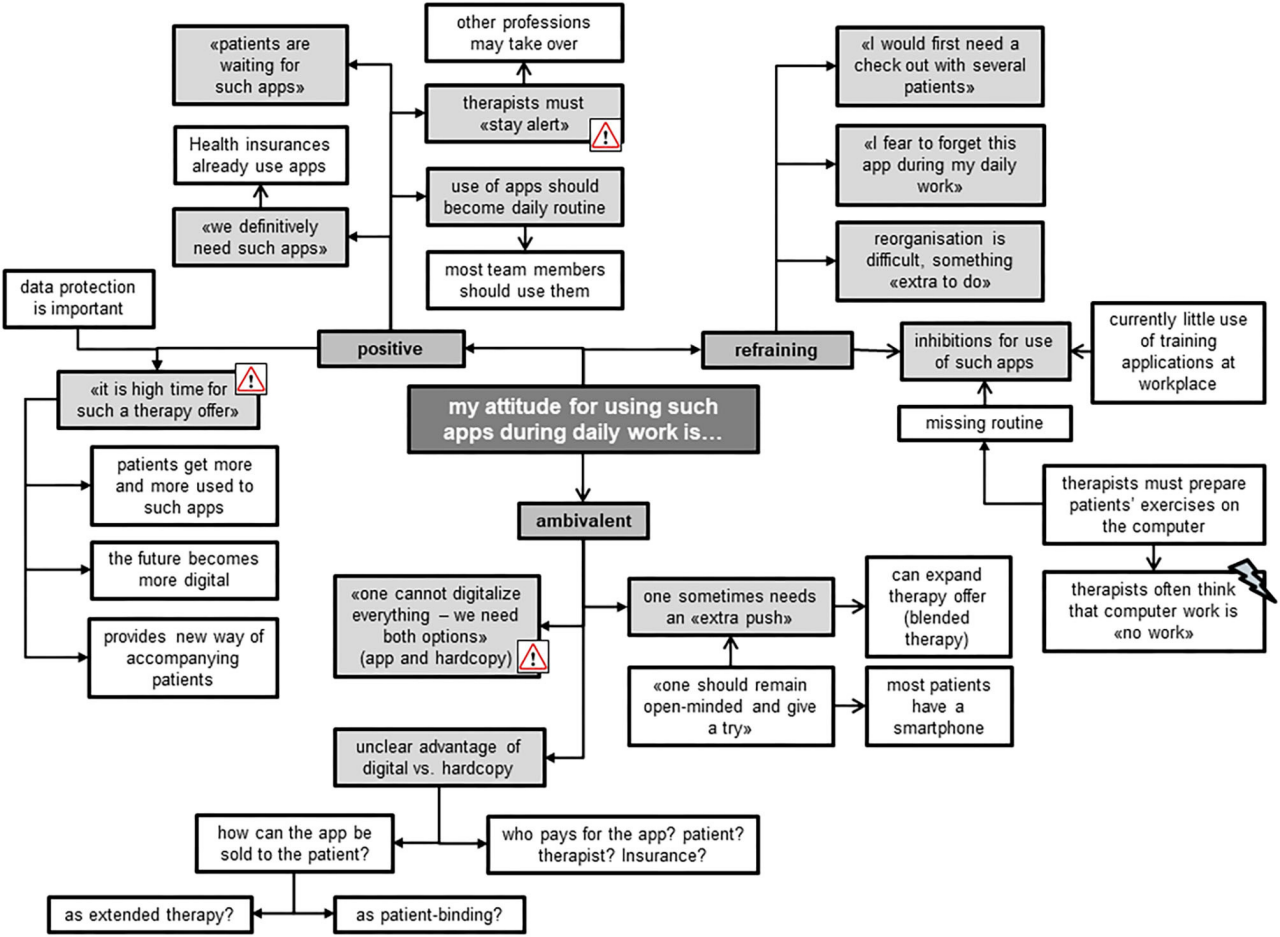
Therapists' attitude for using the Fit app during daily work.

Most of the focus group members shared a positive attitude toward using such apps for therapeutic purposes (Figure 8). They all agreed that therapists should stay alert to new therapy developments. To date, they described mostly using hardcopies to compose individualized home exercise programs for their patients, together with the smartphone of the patient to take pictures or make videos. Exercise apps on the computer were rarely used, because they were described as being more time-consuming than using hardcopy training schedules or oral instructions during therapy. The TL highlighted the importance of such apps running smoothly, as technical difficulties often reduce their usability. The protection of data privacy was an important issue for all therapists. Those members of the focus group with a reluctant and ambivalent attitude mentioned that they perceived it difficult to change and adjust to the “digital world.” They stated that they do not perceive computer work as dedicated time to patients, experiencing a barrier to using IT in their presence. One advantage for using the Fit app was seen in its ability to stay up-to-date with patients' progress, while exercises on paper bear the risk of patients still performing exercises at home that have ceased to be effective. Nevertheless, they preferred using both digital and handwritten exercise programs.

## Discussion

Overall, the app has demonstrated to be usable for both therapists and trainees. Effectiveness measured by task completion rate improved from phase one to phase two from 73 to 90% and from 85 to 92% for the “play” and “manager” components, respectively. In contrast, because in both phases there was one task which was not completed within the expected timeframe, efficiency did not change. Whereas, satisfaction with “play” improved (from 70.5 to 81.5), it decreased with “manager” (from 85.5 to 73.5) from phase one to phase two. Overall acceptance with the app showed the same pattern as satisfaction. Acceptance of “play” improved from 111 to 120 points and decreased for “manager” from 101 to 97. Therapists from the focus group interviews perceived this app suitable for different patient groups and appraised it worth trying in their clinical practice.

Although most of the detected errors from phase one could be remedied before phase two started, satisfaction and acceptance improved only for “play” (assessed by trainees) but not for “manager” (assessed by therapists). One reason that satisfaction and acceptance of the “manager” decreased in phase two might be that some errors were only detected in the second phase, although they already existed in the first phase. It seems that therapists from the second phase were more critical than those from the first phase. This interpretation is confirmed by the fact that therapists from phase two made more critical comments during the task “*create an individual exercise program”* than those from phase one. In comparison, the trainees detected no further errors in the second phase and comments were less critical than in phase one. One explanation for this result is that therapists indicated being more familiar with technology than trainees, especially for handling tablet computers. Perhaps, therefore, the former expected less from this app and were thus less critical. Another reason for decreased satisfaction and acceptance of the “manager” in phase two might be that some problems could only be seen in the second phase. For example in the first phase the overview of the training diary was so unclear that therapists did not go into details, but just accepted that this part requires improvement. In phase two, the overview improved slightly, but was still not satisfying. Therapists tried hard to interpret the figures and tables, but since it was still very confusing, they were not successful. This may be more frustrating and therefore they were less satisfied and consequently less convinced that they would use the app.

Although trainees were not able “*to check the training”* (task 11) in “play,” it seems that therapists weight this task as more important than do trainees. For the latter, it was important to understand the exercise and be able to record their training diary. They were not very interested in the overview. Perhaps this was also because they did not perform a real training session but recorded only one virtual one. Therefore, progress could not be seen in the training overview. For the therapists, the training diary was one of the most important tasks of this app. They can only coach their trainees adequately when they have a good overview of the performed training sessions. Therefore, the failure of this task might have a higher impact on satisfaction and acceptance than the failure of task 2 (*enter birthdate of the trainees*). In addition, the fact that SUS as well as TAM-scores were at a high level in the first phase made it difficult to further improve scores in the second phase. SUS scores for example were higher than the described average score of 68 from normative data (42). Scores from therapists even reached the threshold of 82 points, which is important for a system to be recommended to a friend (38).

In general, trainees as well as therapists judge the Fit app as usable and also had a positive attitude toward its use. This is consistent with the overall judgement from the participants of the focus groups. Some points were emphasized in both settings, such as participants preferring videos to photos, wishing to have more interactive tools such a push news or e-mail contact and wanting to have the scope to add additional exercises. In addition, focus group members emphasized the importance of the ease of use of such an app. This aspect was rated as good in the usability part of this study.

Besides the device itself, the focus group members discussed also contextual factors related to the acceptance of innovative digital tools. The results showed that the focus group members liked the app thanks to its blended character in offering a patient (“play”) and therapist (“manager”) station. With the feature of remote support of the patient, this app provides an opportunity of therapy extension between patient's home and clinic, allowing maintenance of contact with patients from wherever they are. Our results corroborate previous studies that reported high acceptance and satisfaction of blended therapy (defined as the combination of face-to-face therapy and remote support via TT) in patients with anterior cruciate ligament reconstruction, multiple sclerosis and hip/knee osteoarthritis (20, 43, 44).

Despite the high acceptance of the Fit app among therapists, they also raised some concerns regarding its use. Barriers were seen in its implementation in the clinic (work processes need to be adjusted; data protection), in the personal attitude of therapists (change management), together with uncertainties regarding payment of costs and invested therapy time. Kloek et al. also revealed some of these concerns: some therapists perceived the evaluated web-based application as an additional burden within the busy work schedule and their fear that this approach could substitute face-to-face sessions (45). Nevertheless, the therapists had diverse ideas for improvements of this app, putting emphasis on revising the many advantages of an app in comparison with non-technological approaches (e.g., using videos instead of photos, including push-news, providing an interface with the clinic's network to exchange data). This is in line with the therapists' feedback during the usability part of this study. Because the Fit app is part of a bigger software program and all functions and features must be compatible with these other applications, it was not possible to insert photos in the “manager” during the usability study. To overcome this failing, a leaflet with the overview of the exercises was given to the therapists. In the meantime, the software engineers developed a solution to include photos of exercises in the “manager.”

As blended therapy is a relatively new therapy option, it is unclear which patient groups could benefit the most from such an approach. One group might be patients with a relatively long rehabilitation period such as for example, anterior cruciate ligament reconstruction. Patients with rare diseases, e.g., inflammatory myositis or hemophilia, could also benefit from blended therapy, as specialized and experienced health professionals are mostly available in major health centers only. Therefore, access to high quality care is limited by travel distance and travel costs. In this situation, the blended therapy approach may optimize the timing, intensity and sequencing of interventions and provide opportunities for individuals to receive specialized care rehabilitation in their own social and professional environments (46). A recently conducted RCT reported blended therapy to be equally effective as usual physical therapy with respect to physical functioning and free-living physical activity. Both interventions led to similar clinical improvements (21). Results of blended therapy and usual care were also comparable with respect to cost. While intervention costs of a blended therapy approach are significantly lower when compared to usual therapy, overall societal and healthcare costs were not significantly different (47). Therefore, the authors concluded that blended therapy could be used as a suitable alternative for usual care. While digital health options do not interest all patients and not all patients are suitable for a digital tool, decisions about which intervention should be used can be based on patients' preferences, as well as prerequisites such as sufficient internet skills, technology affinity and self-discipline (20, 44, 47).

Our study also had some limitations. First, trainees received a virtual exercise program and had to imagine doing the exercises and then record the virtually performed training parameters. For some trainees, it would have been easier to record an effectively performed training session. However, this would have been too time consuming for this usability study. Second, the app is part of a blended intervention in which therapists examine their trainees, design an individual exercise program and instruct the Fit app for each trainee within one or two face-to-face sessions. Only after these face-to-face sessions can trainees then start their individual program at home. This usability study did not evaluate the interaction between therapists and trainees in the blended intervention process but only the handling of the app in a laboratory setting. Third, the results should be considered with caution because of the small sample size of five end-users within each phase. Nevertheless, tests with five participants are usually able to uncover two thirds of usability issues (29). As we included five trainees and therapists in each phase, most of the usability problems are likely to have been revealed. Fourth, combining our results with other usability methods such as heuristic evaluation or cognitive walkthrough would have strengthened our results (24). As we only had limited financial and personal resources for the development of this app and because we considered the opinion of end-users as the most important benefit, we decided to apply a user-based testing method. Only with the involvement of end-users in the development can users' perspectives such as their needs, expectations, problems and attitudes be considered (48). Finally, the first and second authors of this article conducted the focus group interviews. This might have influenced the participants' expressions of opinions in two ways: firstly, the authors and participants knew each other from daily work, which might have hindered some of the latter to issue critical statements. Secondly, as the first author was also one of the joint inventors of the Fit app, participants might have felt disinclined to fully express their opinions, for fear of causing offense. As the first authors of this study were aware of this bias, they addressed the issue before the interviews, highlighting that all opinions were welcome.

A strength of this study was the two-phase approach for the usability part, whereby end-users could be involved in the stepwise development process and adaptions of the first prototype of the app could be evaluated directly within the next phase. Another strength is the combination of usability testing with the evaluation of the opinions of therapists. Therapists have an important role as facilitator—or if they are insufficiently engaged—as preventer of the usage of such tools (20, 44). Therefore, for successful implementation of this app within daily clinical business, not only ease of use, but opinions of therapists and contextual factors have also to be considered. So far, blended therapy is not a standard approach in hospital settings, therefore it is important to learn what requirements are necessary to implement it in clinical practice.

Although the Fit app is relatively low-key and various ideas for upgrading functions of the app were suggested, end-users judged it as usable. In particularly, the blended therapy approach was perceived as a promising therapy development. In order that implementation of this approach will be successful, clearly defined work processes are needed and it has to be clarified how costs of this innovative therapy option will be managed. Before the app can be used in daily practice, feasibility of this blended therapy approach in a clinical setting should be evaluated.

## Data Availability Statement

All datasets generated for this study are included in the article/supplementary material.

## Ethics Statement

The studies involving human participants were reviewed and approved by Research Ethics Committee of ETH Zurich, Switzerland (protocol number EK 2017-N-27). The patients/participants provided their written informed consent to participate in this study.

## Author Contributions

PB: was responsible for the conception and the acquisition of the data, made the analysis, and wrote the manuscript. BT-A: made substantial contributions to conception and design, collection, analysis and interpretation of qualitative data, and was involved in writing the manuscript critically. EB: made substantial contributions to conception and design and was involved in revising the manuscript critically. RB: made substantial contributions to conception and design and was involved in revising the manuscript critically. RK: made substantial contributions to conception and design and was involved in revising the manuscript critically. All authors read and approved the final manuscript.

## Conflict of Interest

EB was a co-founder of Dividat; the spin-off company that developed the exergame platform used in this study, and is associated to the company as an external advisor. No revenue was paid (or promised to be paid) directly to EB or his institution over the 36 months prior to submission of the work. The remaining authors declare that the research was conducted in the absence of any commercial or financial relationships that could be construed as a potential conflict of interest.

## Abbreviations

app: application
ETH: Eidgenössische Technische Hochschule
RT: resistance training
TT: telecommunication technologies
SUS: system usability scale
TAM: Technology Acceptance Model
TL: team leaders
TM: team members
USZ: University Hospital Zurich.
